# Dual role of acetaminophen in promoting hepatoma cell apoptosis and kidney fibroblast proliferation

**DOI:** 10.3892/mmr.2014.2085

**Published:** 2014-03-28

**Authors:** YUNG-LUEN YU, GIOU-TENG YIANG, PEI-LUN CHOU, HSU-HUNG TSENG, TSAI-KUN WU, YU-TING HUNG, PEI-SHIUAN LIN, SHU-YU LIN, HSIAO-CHUN LIU, WEI-JUNG CHANG, CHYOU-WEI WEI

**Affiliations:** 1 Graduate Institute of Cancer Biology and Center for Molecular Medicine, Taichung 404, Taiwan, R.O.C.; 2 The Ph.D. Program for Cancer Biology and Drug Discovery, China Medical University, Taichung 404, Taiwan, R.O.C.; 3 Department of Biotechnology, Asia University, Taichung 413, Taiwan, R.O.C.; 4 Department of Emergency Medicine, Taipei Tzu Chi Hospital, Buddhist Tzu Chi Medical Foundation, New Taipei 231, Taiwan, R.O.C.; 5 Department of Emergency Medicine, School of Medicine, Tzu Chi University, Hualien 970, Taiwan, R.O.C.; 6 Division of Allergy-Immunology-Rheumatology, Department of Internal Medicine, Saint Mary’s Hospital Luodong, Yilan 265, Taiwan, R.O.C.; 7 Department of Internal Medicine, School of Medicine, College of Medicine, Taipei Medical University, Taipei 110, Taiwan, R.O.C.; 8 Division of General Surgery, Taichung Hospital, Ministry of Health and Welfare, Taichung 403, Taiwan, R.O.C.; 9 Division of Renal Medicine, Tungs’ Taichung Metroharbor Hospital, Taichung 435, Taiwan, R.O.C.; 10 Department of Nutrition, Master Program of Biomedical Nutrition, Hungkuang University, Taichung 433, Taiwan, R.O.C.; 11 Department of Nursing, Taipei Tzu Chi Hospital, Buddhist Tzu Chi Medical Foundation, New Taipei 231, Taiwan, R.O.C.

**Keywords:** acetaminophen, kidney tubular cell, hepatoma, fibroblasts

## Abstract

Acetaminophen (APAP), is a safe analgesic and antipyretic drug at therapeutic dose, and is widely used in the clinic. However, high doses of APAP can induce hepatotoxicity and nephrotoxicity. Most studies have focused on high-dose APAP-induced acute liver and kidney injury. So far, few studies have investigated the effects of the therapeutic dose (1/10 of the high dose) or of the low dose (1/100 of the high dose) of APAP on the cells. The aim of this study was to investigate the cellular effects of therapeutic- or low-dose APAP treatment on hepatoma cells and kidney fibroblasts. As expected, high-dose APAP treatment inhibited while therapeutic and low-dose treatment did not inhibit cell survival of kidney tubular epithelial cells. In addition, therapeutic-dose treatment induced an increase in the H_2_O_2_ level, activated the caspase-9/-3 cascade, and induced cell apoptosis of hepatoma cells. Notably, APAP promoted fibroblast proliferation, even at low doses. This study demonstrates that different cellular effects are exerted upon treatment with different APAP concentrations. Our results indicate that treatment with the therapeutic dose of APAP may exert an antitumor activity on hepatoma, while low-dose treatment may be harmful for patients with fibrosis, since it may cause proliferation of fibroblasts.

## Introduction

Acetaminophen (APAP), also known as paracetamol, is a safe analgesic and antipyretic agent at therapeutic dose (1). It has been widely applied in the clinic (2–4). In general, an overdose of APAP of 10–15 g can cause serious toxicity and is harmful to the liver and the kidneys (5,6). APAP is easily available and cheap, and thus patients may easily receive an overdose. This is one of the reasons that APAP constitutes the most common cause of self-poisoning in numerous countries (7–9). In order to study APAP overdose-induced liver and acute kidney damage, a number of animal and cell models have been established. Studies in these models have shown that treatment with high doses of APAP (300–2,500 mg/kg) can cause hepatotoxicity and nephrotoxicity *in vivo* (10–14), and doses >0.005 mol/l can induce cytotoxicity on kidney and liver cells (15–20). Previous studies have shown that APAP can induce apoptosis or necrosis on different cell models (14,19,21), and that high-dose APAP treatment can increase oxidative stress, decrease the glutathione level and activate MAPK signaling pathways, resulting in cell cytotoxicity (14,16,20,22–25).

A number of recent studies have indicated that high-dose APAP treatment causes liver and kidney failure (26–28). However, other studies reported that high-dose APAP treatment also exerts anticancer effects. These studies showed that APAP can induce cytotoxicity on neuroblastoma (SH-SY5Y cells), hepatoma (HuH7 cells) and breast cancer (FM3A cells) (29–33). These studies also demonstrated, in different tumor cell types, that APAP-induced cell death is related to the proteins NF-κB, members of the Bcl-2 family, and the glycogen synthase kinase-3. In addition, APAP can enhance the chemotherapeutic anticancer effects of drugs used to treat neuroblastoma, leukemia and ovarian carcinoma (30,34,35). According to the above studies, APAP can activate different cytotoxic mechanisms in liver, kidney and tumor cells (14,19,21,31,36). To date, most studies have focused on the mechanisms of APAP-induced cytotoxicity and on how to prevent high-dose APAP-related poisoning of the liver and the kidneys. However, whether APAP can enhance cell proliferation remains unclear.

Kidney tubular epithelial cell damage can induce renal failure (37–40). Kidney fibrosis, via fibroblast proliferation, can also cause renal failure (41–43). Therefore, both kidney tubular cell damage and fibroblast proliferation can cause kidney dysfunction. Recently, high-dose APAP-induced nephrotoxicity was reported and investigated (13,22,44–47). These studies found that high-dose APAP treatment can induce kidney tubular cell death in animal and cell models. In addition, numerous studies have demonstrated that high-dose APAP treatment can induce an increase in oxidative stress, causing tubular cell death through necrosis or the apoptotic pathway (13,22,44,47,48). However, there is no evidence that APAP can cause kidney dysfunction by inducing fibroblast proliferation. The present study is the first to demonstrate, to the best of our knowledge, that high doses of APAP (7.94 mM) can inhibit cell survival in kidney tubular cells (NRK-52E), while promoting cell proliferation in kidney interstitial fibroblasts (NRK-49F).

In addition, APAP can induce different cytotoxic mechanisms on different hepatoma cell lines. APAP can induce caspase-dependent apoptosis on hepatoma HuH7 and SK-Hep1 cells (31,49) and induces apoptosis and necrosis on hepatoma HepG2 cells (50). Additionally, a study demonstrated that high-dose APAP treatment can inhibit DOX-induced cell death in hepatoma HepG2 cells (36). Although APAP-induced apoptosis of hepatoma Hep3B cells was reported (51), the underlying mechanisms are still unclear.

## Materials and methods

### Materials

Luminol, lucigenin and Hoechst 33342 were purchased from Sigma-Aldrich (St. Louis, MO, USA). Transforming growth factor (TGF)-β was purchased from R&D Systems (Minneapolis, MN, USA). The MTT assay kit was purchased from Bio Basic Canada, Inc. (Markham, ON, Canada). The caspase-9 substrate acetyl-Leu-Glu-His-Asp-*p*-nitroanilide (Ac-LEHD-pNA), the caspase-3-like substrate acetyl-Asp-Glu-Val-Asp-*p*-nitroanilide (Ac-DEVD-pNA) and the caspase-8 substrate acetyl-Ile-Glu-Thr-Asp-*p*-nitroanilide (Ac-IETD-pNA) were purchased from AnaSpec, Inc. (San Jose, CA, USA). Fetal bovine serum (FBS), Dulbecco’s modified Eagle’s medium (DMEM), non-essential amino acids, L-glutamine and penicillin/streptomycin were purchased from Gibco-BRL (Carlsbad, CA, USA).

### Cell lines and cultures

The rat kidney cell lines NRK-52E (tubular epithelial cells) and NRK-49F (fibroblasts) and Hep3B cells were purchased from the Bioresource Collection and Research Center (Hsinchu, Taiwan). These cell lines were cultured in DMEM medium supplemented with 10% FBS, 2 mM L-glutamine, 100 IU/ml penicillin/streptomycin and 0.1 mM non-essential amino acids, and were maintained at 37°C in a humidified atmosphere containing 5% CO_2_, as in (52,53).

### Cell survival assay

Survival rates of NRK-52E, NRK-49F and Hep3B cells were determined with the MTT assay as previously described (54,55). Briefly, cells were cultured in 96-well plates. On the second day, cells were divided into the control and experimental groups. After cells were treated with 7.94 nM APAP, 0.794 nM APAP, 0.0794 nM APAP and 1nM TGF-B, respectively, cell survival rates were measured every day. The MTT assay was conducted daily according to the manufacturer’s instructions. Absorbance was measured at 570 nm using a multi-well ELISA reader (Molecular Devices, Sunnyvale, CA, USA).

### Quantification of H_2_O_2_ and O_2_^−^ levels

H_2_O_2_ and O_2_^−^ levels were measured using a lucigenin-amplified chemiluminescence method, as in (56,57). Briefly, 200 μl of cell lysate was mixed with 0.2 mmol/l of luminol solution (100 μl) for the quantification of the H_2_O_2_ level, or with 0.1 mmol/l of lucigenin solution (500 μl) for the quantification of the O_2_^−^ level. Measurements were then performed on the CLA-FSI chemiluminescence analyzing system (Tohoku Electronic Industrial Co., Ltd., Sendal, Japan). Each assay was performed four times and results were expressed as the chemiluminescence count per 10 sec.

### Nuclear observation

Nuclear morphology was observed by nuclear staining with Hoechst 33342. Cells were treated with Hoechst 33342 (10 μg/ml) for 10 min. Nuclear condensation and DNA fragmentation were observed under a fluorescence microscope (excitation, 352; emission, 450 nm; Olympus BX61; Olympus Corporation, Tokyo, Japan), as described in previous studies (58,59).

### Caspase activity assay

Cells were treated with lysis buffer (50 mM Tris-HCl, 120 mM NaCl, 1 mM EDTA, 1% NP-40, pH 7.5), and then 1 μM protease inhibitors (Cocktail set 539131; Merck KGaA, Darmstadt, Germany) were added. Cell pellets were obtained by centrifugation (15,000 × g, 4°C, 30 min). Caspase-3, -8 and -9 activities were determined based on assays described in previous studies (60–62). Briefly, 40 μl of cell lysate (80 μg total protein) were mixed with 158 μl reaction buffer (20% glycerol, 0.5 mM EDTA, 5 mM dithiothreitol, 100 mM HEPES, pH 7.5) and 2 μl fluorogenic substrate (Ac-LEHD-pNA, Ac-DEVD-pNA, or Ac-IETD-pNA) and were incubated at 37°C for 6 h. The absorbance of the cleaved fluorogenic substrate was detected at 405 nm (A405) in a FLx800™ fluorescence microplate reader (BioTek Instruments, Inc., Winooski, VT, USA). The fold increase (FI) in caspase activity was calculated using the following formula: FI = (A405_sample_ − A405_control_)/A405_control_.

### Data analysis

Data were obtained from four independent triplicate experiments and are presented as mean values of all data, with related standard deviations (SD).

## Results

### APAP treatment reduces the survival rate of kidney tubular epithelial cells, while inducing proliferation of kidney fibroblasts

**Figure 1:**
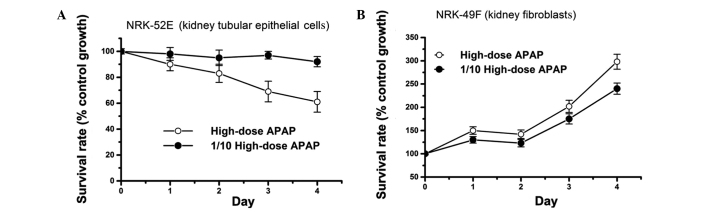
Effects of high-dose acetaminophen (APAP) treatment on the survival rates of the kidney cell lines (A) NRK-52E and (B) NRK-49F. Cells were treated with a high dose and 1/10 of the high dose of APAP. Survival rates were calculated daily using the MTT assay. Data are presented as mean ± SD from four independent experiments.

Previous studies showed that a high dose of APAP (>5 mM) can cause cell cytotoxicity *in vitro* (15–20). In accordance with these studies, we also found that high-dose (7.94 mM) APAP treatment reduces the survival rate of kidney tubular epithelial cells (NRK-52E line), in a time-dependent manner (Fig. 1A). The survival rate of NRK-52E cells did not decrease upon treatment with 1/10 of the high dose of APAP compared to high-dose treatment (Fig. 1A). These results suggest that APAP-induced cell cytotoxicity is dependent on APAP concentration and incubation time. However, to our surprise, although high-dose APAP treatment decreased the survival rate of NRK-52E cells, it promoted cell proliferation of kidney fibroblasts (NRK-49F line) (Fig. 1B). This was also observed upon treatment with 1/10 of the high dose of APAP (Fig. 1B). It is well established in the clinic that both tubular epithelial cell damage and kidney fibrosis can induce renal failure (13,22,41,43,47,48,63,64). Therefore, our findings indicate that APAP-induced renal failure may not only relate to the inhibition of tubular epithelial cell survival, but also to the promotion of renal fibroblast proliferation.

### Low-dose APAP treatment induces proliferation of kidney fibroblasts

**Figure 2:**
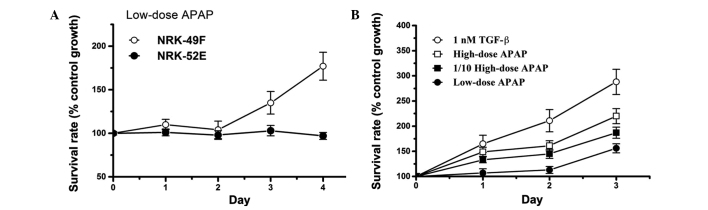
Effects of low-dose acetaminophen (APAP) treatment on kidney cell survival rates. Survival rates were calculated daily using the MTT assay. (A) NRK-49F and NRK-52E cells were treated with a low dose of APAP. (B) NRK-49F cells were treated with a high dose, 1/10 of the high dose and a low dose of APAP, as well as with the transforming growth factor (TGF)-β, as a positive control. Data are presented as mean ± SD from four independent experiments.

Previous studies have demonstrated that high-dose APAP treatment can inhibit tubular epithelial cell survival to induce renal failure (13,22,43,47,48). In the present study, as shown in Fig. 1, high-dose APAP treatment inhibited growth of tubular epithelial cells, and induced proliferation of kidney fibroblasts. In patients with kidney fibrosis, it is important to prevent fibroblast proliferation, which further aggravates their condition. In order to enhance our understanding on the effects of APAP treatment on patients with fibrosis, it is therefore valuable to investigate whether low doses of APAP (below the therapeutic dose) can induce fibroblast proliferation. In this study, low-dose APAP treatment was applied on kidney fibroblasts to study its effects on cell growth. It is notable that low-dose APAP treatment did not inhibit cell survival of NRK-52E cells, while low-dose treatment induced cell proliferation in the fibroblast cell line NRK-49F (Fig. 2A). In addition, APAP induced fibroblast proliferation similarly to the treatment with the positive control TGF-β, and in a dose-dependent manner (Fig. 2B). APAP has not been reported to be toxic to liver and kidney cells at doses below the therapeutic dose in the clinic. However in our experiments, a low dose of APAP induced fibroblast proliferation, which may have harmful effects in patients with fibrosis. Thus, our study suggests that these patients may be sensitive to even low doses of APAP.

### The cytotoxic effects of APAP are more prominent in Hep3B compared to NRK52 cells

**Figure 3:**
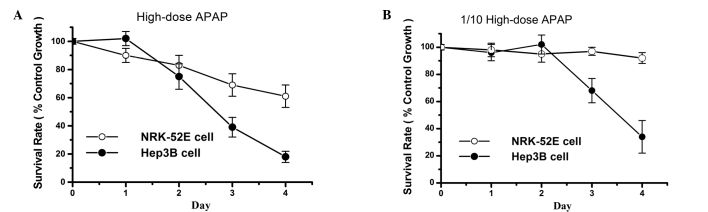
Effects of acetaminophen (APAP) on hepatoma cell survival rates. Survival rates were calculated daily using the MTT assay. (A) NRK-52E and Hep3B cells were treated with a high dose of APAP. (B) NRK-52E and Hep3B cells were treated with 1/10 of the high dose of APAP. Data are presented as mean ± SD from four independent experiments.

High-dose APAP treatment induced cytotoxic effects not only in the tubular cell line NRK-52E, but also in the hepatoma cell line Hep3B (Fig. 3A). Cell survival rates of treated Hep3B cells were lower compared to those observed in NRK-52E cells. However, at an APAP concentration that was 1/10 of the high dose (therapeutic dose), no obvious cytotoxic effects were observed in NRK-52E cells, while the survival rate of Hep3B cells was markedly reduced (Fig. 3B). Therefore, APAP exerts more prominent cytotoxic effects on Hep3B compared to NRK-52E cells. These results indicate that APAP, at a non-toxic concentration for healthy tubular cells, may exert an antitumor effect on hepatoma cells.

### APAP treatment increases apoptosis of Hep3B cells via an increase in the H_2_O_2_ level

**Figure 4:**
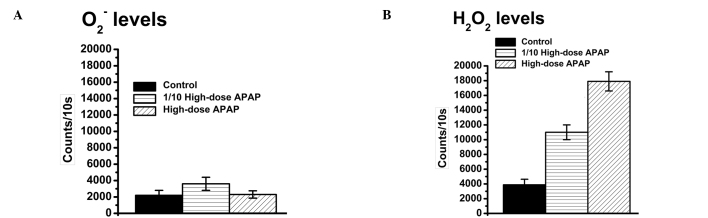
Effects of acetaminophen (APAP) on (A) O_2_^−^ and (B) H_2_O_2_ levels in Hep3B cells. Measurements were performed following APAP treatment (6 h) using a lucigenin-amplified chemiluminescence method. Control (non-treated), high-dose APAP-treated and 1/10 high-dose APAP-treated cells were examined. Data are presented as mean ± SD from four independent experiments.

**Figure 5:**
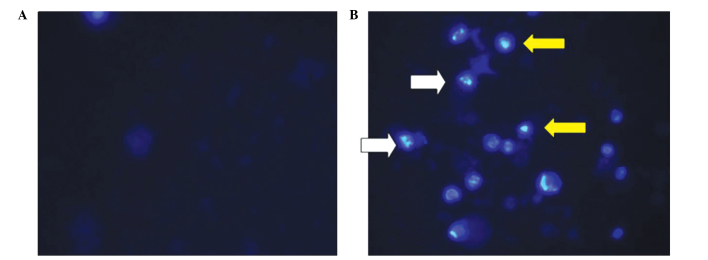
Effects of acetaminophen (APAP) treatment on nuclear condensation and DNA fragmentation. (A) Control (non-treated) and (B) APAP-treated Hep3B cells. Following cell treatment with APAP for 72 h, nuclear morphology was observed by nuclear staining with the Hoechst 33342 dye. Nuclear condensation (yellow arrows) and DNA fragmentation (white arrows) were observed on APAP-treated cells.

APAP-induced cytotoxic effects that relate to an increase in the generation of reactive oxygen species (ROS) were previously reported (65,66). However, it is still unclear which ROS elements are increased upon APAP treatment. O_2_^−^ and H_2_O_2_ are two commonly found ROS types in the cells. O_2_^−^ and H_2_O_2_ levels were thus quantified following APAP treatment. The result showed that APAP causes an increase in the H_2_O_2_ (Fig. 4A), but not in the O_2_^−^, level in Hep3B cells (Fig. 4B). Therefore, APAP-induced cytotoxicity is possibly related to H_2_O_2_ but not to O_2_^−^. In addition, microscopic observations of the nuclear morphology revealed nuclear condensation and DNA fragmentation in the APAP-treated Hep3B cells (Fig. 5). These results overall suggest that APAP can induce cell cytotoxicity via an increase in the H_2_O_2_ level.

### APAP activates the caspase-9/-3 cascade in Hep3B cells

**Figure 6:**
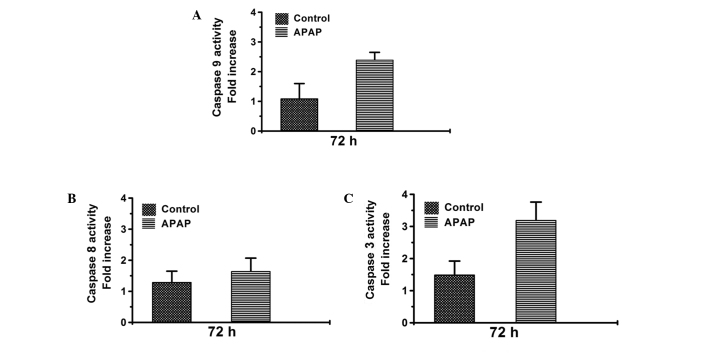
Activity of (A) Caspase-9; (B) caspase-8; and (C) caspase-3 activities in control (non-treated) and acetaminophen (APAP)-treated cells. Caspase-3 and -9 activities are increased in cells treated with 1/10 of the high dose of APAP. Data are presented as mean ± SD from three independent experiments.

Caspase activation can induce cell apoptosis (60,61). In our study, APAP treatment also induced apoptosis of Hep3B cells, as indicated by results presented in Figs. 4 and 5. Therefore, caspase activities were next measured in Hep3B cells, focusing on the two major caspase cascades, the caspase-9/-3 and the caspase-8/-3, and using a substrate cleavage assay as previously described (60,61). The caspase-9 and -3 activities were found induced by treatment with 1/10 of the high dose of APAP (Fig. 6A and C) However, the caspase-8 activity did not notably change upon APAP treatment (Fig. 6B). This result suggests that APAP can activate the caspase-9/-3 cascade to induce cell cytotoxicity in Hep3B cells.

## Discussion

Both tubular epithelial cell damage and fibroblast proliferation can induce renal dysfunction (13,22,41,43,47,48,63,64). Numerous studies have demonstrated that an APAP overdose can reduce tubular epithelial cell survival, resulting in nephrotoxicity (13,22,43–46). Most of the studies to date have focused on high-dose APAP-induced acute intoxication of kidney tubular cells. These studies have highlighted the need to further investigate the effects of APAP and take these effects into consideration in order to prevent APAP-induced acute damage. However, it is still unclear whether low doses of APAP may cause chronic kidney damage. Our present study demonstrated that high-dose APAP treatment not only reduces survival of tubular epithelial cells, but it can also induce proliferation of fibroblasts, even at low doses. This implies that APAP-induced renal damage may occur through epithelial cell damage or fibroblast proliferation. In general, acute damage is easier to detect and diagnose compared to chronic damage; therefore, APAP overdose-induced acute intoxication is commonly observed, whereas low-dose APAP-induced damage is more likely to be ignored in the clinic. Here, we demonstrated that low-dose APAP treatment can promote fibroblast proliferation. Thus, we consider the therapeutic dose of APAP to be a safe analgesic and antipyretic agent for patients who do not show fibrosis, but potentially harmful to patients with kidney fibrosis.

The TGF-β signaling pathway was shown to be involved in renal damage (67–69). TGF-β-induced renal damage has been associated with: i) tubular cell death (68,70,71); ii) epithelial mesenchymal transition (72,73); and iii) fibroblast proliferation (74,75). Up to now, no study has provided evidence that APAP can induce kidney fibroblast proliferation via TGF-β-related signals. In this study, NRK-49F cells (fibroblasts) treated with APAP showed a similar induction in proliferation to the one observed in the group treated with TGF-β. In addition, a previous study showed that TGF-β is significantly elevated in APAP-treated liver tissue (71). Based on these observations, we hypothesize that APAP induces kidney fibroblast proliferation via the TGF-β signaling pathway. Whether APAP also exerts effects on epithelial mesenchymal transition in kidney tubular cells warrants future investigation.

O_2_^−^ and H_2_O_2_ are two commonly found ROS in the cells. They are typically produced by the electron transport chain. O_2_^−^ can be removed from the cells through the enzymatic activity of superoxide dismutase, and H_2_O_2_ through the activity of catalase or glutathione. It is well established that cell damage occurs when O_2_^−^ and H_2_O_2_ levels are increased. Previous studies showed that an APAP overdose can increase ROS levels and eventually, reduce cell viability (51,76). However, these studies did not directly demonstrate which ROS element is increased upon APAP treatment. Here, two types of ROS (O_2_^−^ and H_2_O_2_) were quantified following APAP treatment. The H_2_O_2_ level increased, but no notable change in the O_2_^−^ level was observed in APAP-treated cells. Our study suggests that the inhibition of cell survival by APAP may occur through an increase in the H_2_O_2_ level. This is possibly the reason why N-acetyl cysteine, a substrate for glutathione synthesis, is applied on patients with APAP-induced poisoning in emergency clinical cases (77,78).

APAP-induced cell death has been extensively studied (13,22,44,47,48). These studies demonstrated that APAP induces cell death either via the apoptotic or the necrotic death pathways in different cells. In our study, features of apoptosis were observed in APAP-treated Hep3B cells, similar to previous studies (51,75). Moreover, our study further demonstrated that the caspase-9/-3 cascade is activated upon APAP treatment, while the caspase-8/-3 cascade is not. Caspase-9/-3 signaling related to mitochondrial damage and caspase-8/-3 signaling related to death receptor signals have been previously reported (60,61). Thus, our data suggest that APAP-induced cell cytotoxicity might be associated with mitochondrial damage in Hep3B cells. Finally, previous studies have shown cytotoxicity upon high-dose (>5 mM) APAP treatment *in vitro* (15–20). In this study, high-dose APAP treatment induced cytotoxicity in both healthy kidney tubular cells and hepatoma cells. However, 1/10 of this dose was only cytotoxic to hepatoma cells. This suggests that non-toxic (to healthy cells) doses of APAP may be applied in the future as antitumor agents targeting cancer cells.

In summary, the present study shows that: i) APAP treatment can induce cell proliferation of kidney fibroblasts even at low doses, and thus we suggest that APAP treatment needs to be carefully monitored in patients with fibrosis; ii) APAP treatment can increase the H_2_O_2_ level and activate the caspase-9/-3 cascade to cause cytotoxicity; and iii) the cytotoxic effects of APAP depend on the cell type, with hepatoma cells being more severely affected compared to healthy kidney tubular cells.
